# Bioinformatics study of the potential therapeutic effects of ginsenoside Rh3 in reversing insulin resistance

**DOI:** 10.3389/fmolb.2024.1339973

**Published:** 2024-05-23

**Authors:** Yayun Wang, Dongming Wu, Yongxin Wang, Jingwen Sun, Xiaona Wang, Yanqin Huang, Mingliang Sun

**Affiliations:** ^1^ Department of Neurology, Qingdao Traditional Chinese Medicine Hospital (Qingdao Hiser Hospital) Qingdao Hiser Hospital Affiliated of Qingdao University, Qingdao, Shandong, China; ^2^ Department of Geriatric Medicine, Affiliated Hospital of Shandong University of Traditional Chinese Medicine, Jinan, Shandong, China; ^3^ Intensive Care Unit II, Qingdao Traditional Chinese Medicine Hospital (Qingdao Hiser Hospital) Qingdao Hiser Hospital Affiliated of Qingdao University, Qingdao, Shandong, China; ^4^ College of Acupuncture and Massage, Beijing University of Chinese Medicine, Beijing, China; ^5^ Department of Nephrology, Affiliated Hospital of Shandong University of Traditional Chinese Medicine, Jinan, Shandong, China; ^6^ Department of Endocrinology, Affiliated Hospital of Shandong University of Traditional Chinese Medicine, Jinan, Shandong, China

**Keywords:** ginsenoside Rh3, insulin resistance, bioinformatics analysis, metabolic syndrome, molecular docking

## Abstract

**Background:**

In recent years, the incidence of insulin resistance is increasing, and it can cause a variety of Metabolic syndrome. Ginsenosides have been clinically proven to improve fat metabolism and reduce insulin resistance, but their components and mechanism of action are still unclear.

**Objective:**

Ginsenoside, a bioactive compound derived from ginseng, exhibits significant potential in treating obesity, diabetes, and metabolic disorders. Despite evidence supporting its efficacy in ameliorating insulin resistance (IR) in obesity, the specific bioactive components and underlying mechanisms remain obscure. In this study, we endeavored to elucidate the potential molecular targets and pathways influenced by ginsenoside Rh3 (GRh3) to ameliorate IR in liver tissue. We employed a comprehensive approach that integrates system pharmacology and bioinformatics analysis.

**Materials and methods:**

Our methodology involved the identification of candidate targets for GRh3 and the profiling of differentially expressed genes (DEGs) related to IR in individuals with insulin resistance. The coalescence of candidate targets and DEGs facilitated the construction of a “GRh3-targets-disease” network for each tissue type, ultimately yielding 38 shared target genes. Subsequently, we conducted pathway enrichment analysis, established protein-protein interaction (PPI) networks, and identified hub targets among the GRh3 targets and IR-related DEGs. Additionally, we conducted animal experiments to corroborate the role of these hub targets in the context of GRh3.

**Results:**

Our investigation identified a total of 38 overlapping targets as potential candidates. Notably, our analysis revealed crucial hub targets such as EGFR, SRC, ESR1, MAPK1, and CASP3, alongside implicated signaling pathways, including those related to insulin resistance, the FoxO signaling pathway, the PPAR signaling pathway, and the IL-17 signaling pathway. This study establishes a robust foundation for the mechanisms underlying GRh3’s efficacy in mitigating IR. Furthermore, these results suggest that GRh3 may serve as a representative compound within the ginsenoside family.

**Conclusion:**

This study elucidates the potential molecular targets and associated pathways through which GRh3 ameliorates IR, showcasing its multifaceted nature, spanning multiple targets, pathways, and mechanisms. These findings establish a robust foundation for subsequent experimental inquiries and clinical applications.

## 1 Introduction

Insulin resistance (IR) is characterized by a dysregulation in insulin-mediated control of glucose metabolism across various tissues, including muscle, adipose, and hepatic tissues. This physiological disruption is intricately linked with the onset and progression of metabolic syndrome, a cluster of conditions including obesity, type 2 diabetes, and non-alcoholic fatty liver disease (NAFLD), which collectively pose significant health challenges to our society. Epidemiological studies underscore the alarming rise of metabolic disorders as a leading cause of mortality globally (Zimmet et al., 2001).

The liver, as a pivotal metabolic organ, has long been recognized for its central roles in lipid metabolism and insulin regulation (Watt et al., 2019). Aberrant hepatic insulin action is postulated to be a key driver of insulin resistance. While insulin typically fosters anabolic metabolism in the liver by stimulating glucose uptake and lipid synthesis, individuals with insulin resistance exhibit impaired suppression of hepatic glucose production and, paradoxically, heightened liver lipid synthesis. This aberration culminates in elevated circulating levels of glucose and triglycerides, leading to hyperglycemia and hypertriglyceridemia. Consequently, compensatory increases in circulating insulin levels are necessitated to maintain blood glucose homeostasis in the face of insulin resistance. However, this compensatory mechanism ultimately precipitates pancreatic β-cell dysfunction, contributing to the constellation of metabolic syndrome (Muoio and Newgard, 2015).

Given the profound implications of insulin resistance in the pathogenesis of metabolic syndrome, interventions aimed at ameliorating IR harbor considerable potential to attenuate the progression of metabolic syndrome and mitigate associated health risks, including obesity, type 2 diabetes, and NAFLD.

The underlying biology of metabolic syndrome presents a complex and elusive landscape, with mechanisms driving the development of IR and metabolic dysfunction still incompletely understood. While lifestyle modifications and pharmaceutical interventions serve as cornerstone strategies for preventing and managing metabolic syndrome, the effectiveness of lifestyle changes is frequently impeded by challenges in patient adherence. Consequently, pharmaceutical interventions emerge as a pivotal avenue for treatment. Traditional Chinese Medicine (TCM), revered for its extensive history of precise therapeutic outcomes and minimal adverse effects, has emerged as a significant contender in the realm of preventing and treating diverse manifestations of metabolic syndrome. Within the vast array of TCM modalities, ginseng, a frequently utilized Chinese herbal remedy, has garnered considerable attention. Ginsenoside Rh3 (GRh3), an active compound derived from ginseng, has demonstrated a wide-ranging spectrum of benefits, including anti-cancer properties (Teng et al., 2022), anti-inflammatory effects (Lee et al., 2015), memory enhancement (Kim et al., 2013), endometrial cell protection (Wang et al., 2020), and retinal preservation (Tang et al., 2018). Nevertheless, the potential of GRh3 in ameliorating insulin resistance remains largely untapped. In recent years, high-throughput omics data analysis and network pharmacology have emerged as indispensable tools for elucidating drug targets with precision. This approach has garnered recognition for its efficacy in unraveling the pharmacological mechanisms underlying TCM (Chen et al., 2022). In the present study, we embarked on a comprehensive investigation to identify pivotal targets and signaling pathways influenced by ginsenoside Rh3 using a combination of network pharmacology and molecular docking methodologies. Our primary aim was to illuminate the potential involvement of these targets and pathways in enhancing IR. To substantiate our findings, we conducted experiments employing a high-fat diet (HFD) induced IR mouse model. Our endeavor seeks to uncover the intricate mechanisms through which ginsenoside Rh3 ameliorates IR, thereby contributing to a deeper understanding of its therapeutic efficacy.

## 2 Materials and Methods

### 2.1 Drug target prediction

In our pursuit of potential targets for GRh3, we initiated our investigation by obtaining the molecular structure of GRh3 from the PubChem database. Subsequently, employing a rigorous computational approach, we performed virtual screening utilizing the PharmMapper database (Yuan et al., 2022). Parameters were judiciously configured to exclusively target human protein entities, as stipulated by the criteria “Human Protein Targets Only,” with a stringent threshold of “300 Reserved Matched Targets.” Our selection of potential Rh3 targets was contingent upon achieving a Norm Fit score of ≥0.7 through this meticulous *in silico* procedure.

### 2.2 Differentially expressed genes (DEGs) analysis

In our quest to discern the human genes intricately linked with insulin resistance, we meticulously retrieved pertinent data from the Gene Expression Omnibus (GEO) database (Barrett et al., 2013). Our search query encompassed the terms “insulin resistance” (IR) and “non-insulin resistance” (non-IR), narrowing our focus to the *Homo sapiens* species. Following rigorous scrutiny, we selected the dataset GSE23343, which encompasses both insulin-resistant and non-insulin-resistant individuals, for subsequent analysis. To unveil differentially expressed genes (DEGs) between these two distinct cohorts, we harnessed the computational power of the Limma package within the R software environment. For DEG selection, stringent criteria were applied, necessitating an adjusted *p*-value threshold of <0.05 and a fold change magnitude of ≥2.

### 2.3 IR-related target screening

In our endeavor to pinpoint targets associated with IR, we conducted a comprehensive search across multiple databases, including GeneCards (http://www.genecards.org/), TTD (https://db.idrblab.net/ttd/), DisGeNet (https://db.idrblab.net/ttd/), and HPO (https://hpo.jax.org/app/). Our query utilized “insulin resistance” as the primary keyword. To ensure the selection of highly relevant IR-related targets, we adopted a stringent criterion: targets with a relevance score exceeding the upper quartile were retained. The final selection encompassed targets common to both the candidate drug, Ginsenoside Rh3, and insulin resistance. To facilitate a clear visualization of these IR-related targets associated with Ginsenoside Rh3, we employed the jvenn online tool (http://www.bioinformatics.com.cn/static/others/jvenn/index.html).

### 2.4 PPI network construction

For an in-depth exploration of potential interactions, we harnessed the comprehensive resources of the STRING (Search Tool for the Retrieval of Interacting Genes) database (http://string-db.org/). In the pursuit of high-confidence interactions, we set a stringent threshold, exclusively retaining interactions with a confidence score surpassing 0.4. Our analysis of protein-protein interactions (PPI) was visually represented using Cytoscape 3.8.2. Within the network, nodes symbolized targets, while edges denoted relationships between them. An additional layer of analysis involved the identification of core nodes within the network, characterized by higher-than-median values for Betweenness Centrality, Closeness Centrality, and Degree. These core nodes, thus highlighted, occupy pivotal positions within the predicted target network, boasting greater connectivity and influence. Their further exploration promises valuable insights into critical interactions and mechanisms of action concerning GRh3.

### 2.5 GO and KEGG pathway analysis

To unravel the functional implications of our identified IR-related targets for Ginsenoside Rh3, we turned to the DAVID database (https://david.ncifcrf.gov/). Within this robust resource, we conducted comprehensive Enriched Gene Ontology (GO) biological process and Kyoto Encyclopedia of Genes and Genomes (KEGG) pathway analyses (Huang da et al., 2009). The outcomes of these enrichments were further subjected to visualization through Hiplot (https://hiplot.com.cn/). By rendering the enriched GO biological processes and KEGG pathways in a graphical format, we aimed to facilitate a more accessible and insightful presentation of our findings.

### 2.6 Component-target molecular docking

To anticipate the potential impact of GRh3 on IR, we conducted molecular docking analysis employing AutoDock Vina (1.1.2). In this computational endeavor, we scrutinized the common differentially expressed genes (DEGs) shared by IR and ginsenoside Rh3 to forecast their binding interactions. For the molecular docking simulations, both protein structures (comprising the common DEGs of IR) and ligands (comprising compounds such as inhibitors, antagonists, agonists, or substrates) were sourced from the RCSB Protein Data Bank (PDB) (Burley et al., 2021) and relevant literature. The molecular docking grid spanned a cube of dimensions 50 × 50 × 50 Å, artfully centered on the occupied space of the original ligand, with a grid spacing of 0.375 Å. Subsequent analysis of protein-ligand interactions was meticulously conducted employing computational tools, notably PyMOL and Ligplot software (Riyaphan et al., 2021; Laskowski and Swindells, 2011), yielding insights into the intricate binding interactions that govern the potential effects of GRh3.

### 2.7 Animal experiments

Six-week-old male C57BL/6J mice were purchased from Beijing Charles River Co., Ltd. These mice were housed in a specific pathogen-free (SPF) animal facility, and maintained under a 12-h light/dark cycle with stable temperature and humidity (Wu et al., 2023). All animal experimental procedures were performed based on the guidelines for the Administration of Laboratory Animals issued by the Ministry of Science and Technology of the People’s Republic of China. The experiment obtained approval of the Ethics Committee of Affiliated Hospital of Shandong University of Traditional Chinese Medicine (2023-303).

Following 1 week of diet adaptation, the mice were divided into two groups and had adlibitum access to water and normal chow diet (Protein, 24.02 kcal%; fat, 12.95 kcal%; carbohydrates, 63.03 kcal%, BEIJING KEAO XIELI FEED CO., LTD, China) (*n* = 8/group) and high-fat diet (HFD) (Protein, 20 kcal%; fat, 45 cal%; carbohydrates, 35 kcal%, Research Diets, D12451, United States) (*n* = 16/group). The diets were provided adlibitum for a duration of 4 weeks, and after 4 weeks feeding, the HFD mice was randomly divided into the following two groups: the HFD group (*n* = 8), the GRh3-treated group (i.p, 10 mg/kg/day). In addition to the Control group, other groups continued on the high-fat diet. Body weight was recorded weekly, and euthanasia under anesthesia was performed to collect serum and adipose tissur after 6 weeks administration. All experimental procedures were conducted in accordance with the guidelines set by the Animal Ethics Committee of Affiliated Hospital of Shandong University of Traditional Chinese Medicine and adhered to the ARRIVE guidelines.

### 2.8 Intraperitoneal glucose and insulin tolerance tests (ipGTT and ipITT)

After 4 weeks feeding and 6 weeks drug administration, glucose tolerance tests (ipGTTs) and insulin tolerance tests (ipITTs) were conducted on mice following fasting periods of 16 h and 4 h, respectively. Blood glucose levels were measured at different time points (0, 15, 30, 60, 90, and 120 min) subsequent to intraperitoneal injection of glucose (2 g/kg body weight) or insulin (0.75 U/kg body weight). The blood samples were collected via the tail vein and taken for the measurement of glucose levels by ACCU-CHEK (Roche).

### 2.9 Real-time reverse transcription-polymerase chain reaction (qRT-PCR)

Total RNA was isolated from mice liver tissue with TRIzol Reagent (Invitrogen, Carlsbad, CA, United States) and PrimeScript reagent (TaKaRa, Kusatsu, Japan) was used to reverse transcribe into cDNA according to the manufacturer’s instructions. To analyze the target genes’relative mRNA expression, SYBR Green PCR Master Mix Reagent Kit (Yeasen, Shanghai, China) was used to perform real time qPCR using the Roche 480 detection system. The relative mRNA expression levels were normalized by β-actin, and 2^−△△Ct^ method was performed to calculate the results. The primer sequences used are listed in Table 1.

**TABLE 1:** Primer sequences for qPCR.

Gene	Sequence (5′-3′)	
Mouse *EGFR*	F	GCC​ATC​TGG​GCC​AAA​GAT​ACC
	R	GTC​TTC​GCA​TGA​ATA​GGC​CAA​T
Mouse *GAPDH*	F	AGG​TCG​GTG​TGA​ACG​GAT​TTG
	R	TGT​AGA​CCA​TGT​AGT​TGA​GGT​CA
Mouse *SRC*	F	AGA​TCA​CTA​GAC​GGG​AAT​CAG​AGC
	R	AGA​TCA​CTA​GAC​GGG​AAT​CAG​AGC
Mouse *ESR1*	F	AAT​GAA​ATG​GGT​GCT​TCA​GG
	R	ATA​GAT​CAT​GGG​CGG​TTC​AG
Mouse *MAPK1*	F	ATA​GAT​CAT​GGG​CGG​TTC​AG
	R	CCA​CAG​ACC​AAA​TAT​CAA​TGG​ACT​T

### 2.10 Western blotting

RIPA buffer containing PMSF and phosphatase inhibitor was used to lyse cells to extract total protein. The protein samples were separated by 12.5% gel SDS-PAGE, and then transferred onto a PVDF membrane. The membranes were incubated with the following primary antibodies: Anti-Phospho-EGFR antibody (GB114199-100), Anti-ESR1 antibody (GB111843-100) and Anti-MAPK1 antibody (GB11370-100) were purchased from Servicebio Technology (Wuhan, China); Anti-Phospho-SRC (AP0511) and Anti-Hsp90a antibody (A13501) were purchased from ABclonal (Wuhan, China).

### 2.11 Statistical analyses

All data were expressed as the mean ± SD values. Significant differences between the two groups were carried out by unpaired Student’s t-test or one-way ANOVA analysis through GraphPad Prism 8.0. *p* < 0.05 was considered to indicate statistically significant differences.

## 3 Results

### 3.1 Screening of potential targets of GRh3 against IR

**FIGURE 1:**
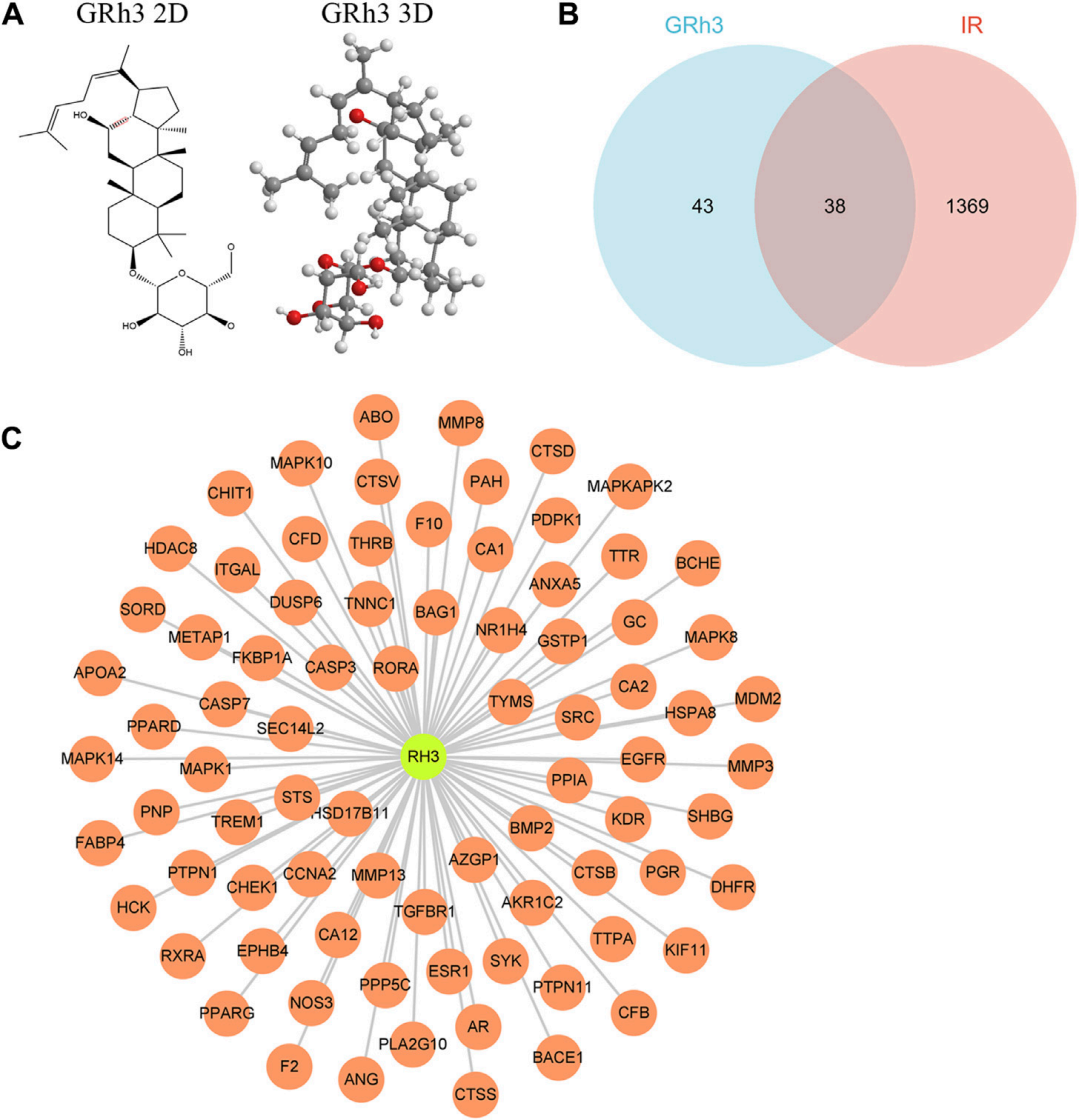
Targets screening involved in GRh3 for treating IR. **(A)** The molecular structure of GRh3. **(B)** Thirt-eight overlapping target proteins between IR-related DEGs and targets of GRh3. **(C)** The relationship between GRh3 and IR-related DEGs.

The molecular structure of ginsenoside Rh3 (GRh3) was extracted from the PubChem database and is illustrated in Figure 1A. We conducted an extensive target identification process using various databases and datasets. PharmMapper database analysis of the GRh3 molecule yielded 81 potential targets. To identify insulin resistance (IR)-related differentially expressed genes (DEGs), we mined the GEO database, specifically dataset GSE23343, retrieving a total of 33 pertinent genes. We performed an exhaustive search across several databases, including Gene Cards, TTD, DisGeNET, and HPO, yielding a cumulative 1,345, 7, 4, and 28 IR-related targets, respectively. Following data integration and removal of duplicate entries, a total of 1407 IR-related DEGs were delineated. Upon amalgamating the identified targets for GRh3 and IR-related genes, we discovered a set of 38 overlapping targets with potential relevance (as depicted in Figure 1B). To elucidate the intricate relationship between GRh3 and IR-related DEGs, we employed Cytoscape 3.8.2 software for network visualization, rendering the connections between these entities visually accessible (Figure 1C).

### 3.2 Visual analysis of the targets of “GRh3-IR”

**FIGURE 2:**
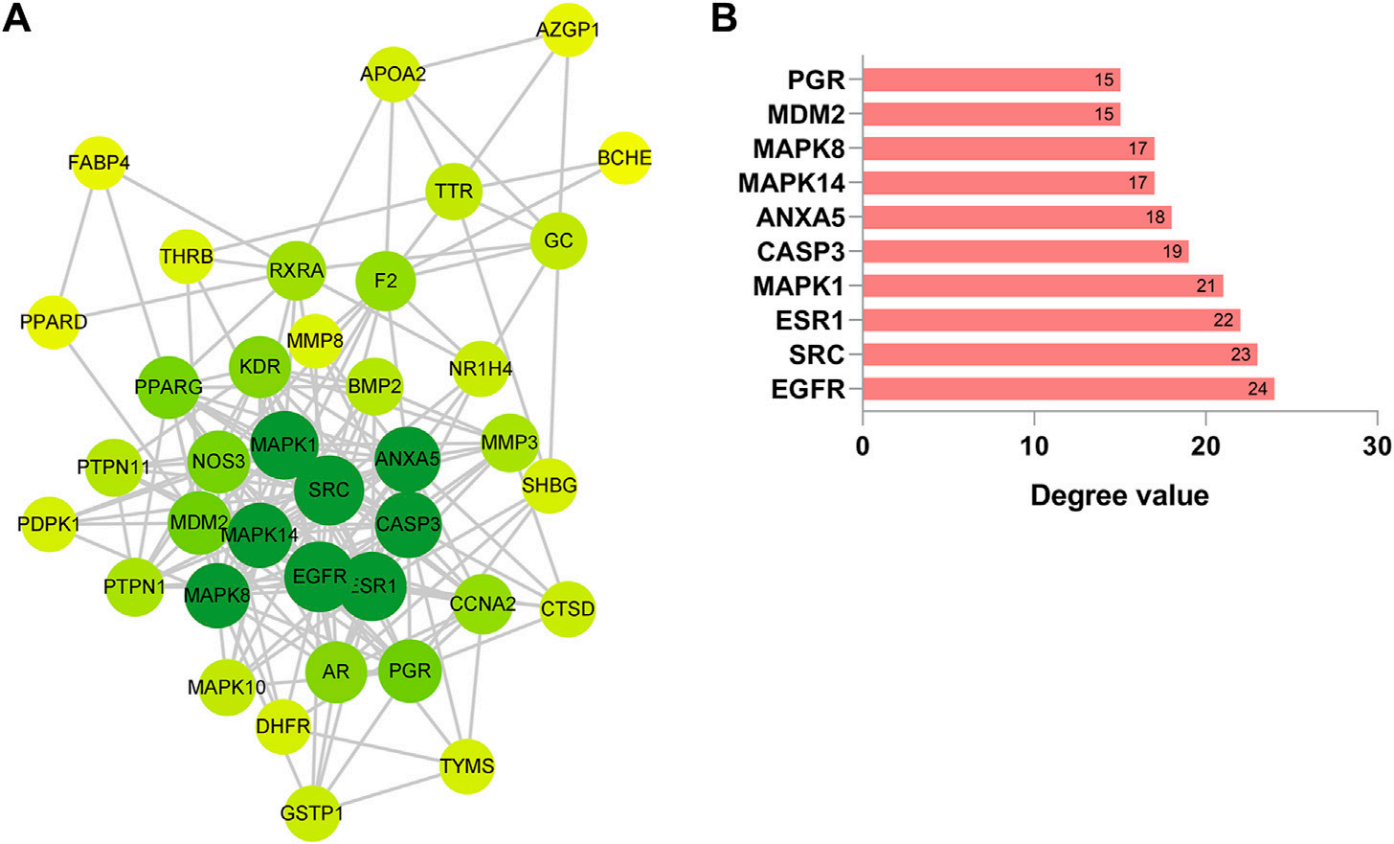
Construction of PPI network. **(A)** PPI network of 38 cross-target. The darker the node color (green), the stronger the connection. **(B)** Most significant module identified by the topology selection.

To delve deeper into the intricate interplay between the drug, ginsenoside Rh3 (GRh3), and the disease, we harnessed the power of network biology. Our initial step involved the utilization of the STRING database to construct a Protein-Protein Interaction (PPI) network based on the 38 overlapping targets (as illustrated in Figure 2A). This network, rendered with precision using Cytoscape software, encompassed 38 target nodes interlinked by 197 edges, offering compelling evidence of the stable binding between GRh3 and the insulin resistance disease targets. Further illuminating this network’s intricacies, we executed a topological analysis, a pivotal step in discerning the most influential nodes within the PPI network. The abscissa in Figure 2B signifies the degree value of each target. Through this analysis, we successfully pinpointed the top 10 key target genes, shedding light on the critical molecular players in GRh3’s mechanism of action.

### 3.3 GO and KEGG pathway enrichment analysis

**FIGURE 3:**
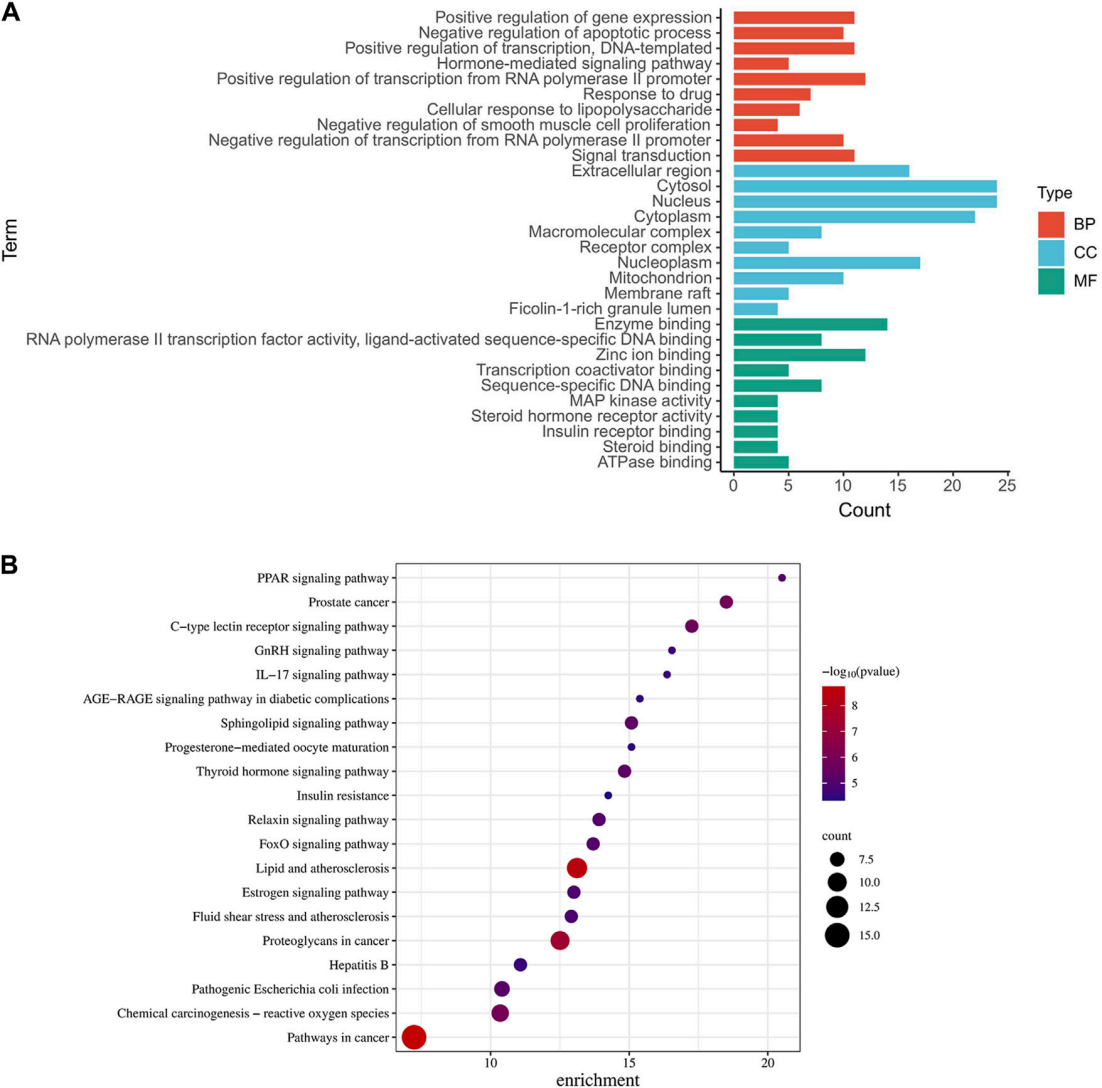
GO and KEGG pathway analysis of intersecting genes. **(A)** The top 10 of GO-BP, GO-CC, and GO-MF enrichment. **(B)** The top 20 of bubble diagram of KEGG pathway enrichment.

To unravel the intricate pharmacological mechanisms orchestrated by ginsenoside Rh3 (GRh3) in insulin resistance, we harnessed the analytical power of the DAVID database. A comprehensive Gene Ontology (GO) analysis of the 38 cross-target genes was conducted, elucidating their involvement in a spectrum of biological processes (BP), molecular functions (MF), and cellular components (CC). This analysis revealed a staggering 205 biological processes, 60 molecular functions, and 28 cellular components impacted by GRh3 (as depicted in Figure 3A). The selection of the top 10 entries from each category, based on their False Discovery Rate (FDR) values, further accentuated the pivotal biological dimensions influenced by GRh3.

To unveil the intricate landscape of pathways influenced by the identified targets, we embarked on a Kyoto Encyclopedia of Genes and Genomes (KEGG) analysis. This analysis not only elucidated the pathways associated with these targets but also spotlighted their relevance to insulin resistance. From a plethora of options, we carefully curated the top twenty KEGG pathways, each seamlessly linked to specific targets (Figure 3B).

In our graphical representation, the size of the icons for pathways and targets corresponded to their significance in the context of medication’s role in disease management. Notably, we identified several noteworthy pathways, including insulin resistance, endocrine resistance, the FoxO signaling pathway, the PPAR signaling pathway, and the IL-17 signaling pathway, all of which emerged as potential focal points for further exploration.

### 3.4 Results of molecular docking of key targets and GRh3

We conducted molecular docking analyses involving the top 10 targets based on degree scoring and the 38 overlapping targets. Utilizing AutoDockTools 1.5.6 software, we facilitated the interaction of small molecule active ligands with their respective large molecule receptor proteins.

**FIGURE 4:**
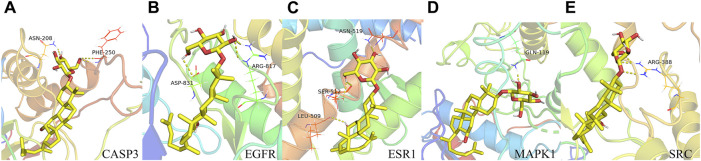
Binding pattern of GRh3 with IR-related targets shown as the 3D representation of 416 interactions. **(A)** CASP3, **(B)** EGFR, **(C)** ESR1, **(D)** MAPK1, and **(E)** SRC.

The outcomes of these molecular docking experiments unveiled critical insights. Notably, the binding energies spanned a range from a maximum of −8.5 kcal/mol to a minimum of −9.2 kcal/mol, as detailed in Table 2 and visually represented in Figure 4. It is imperative to underscore that lower binding energy values signify more efficient and stable interactions between the ligands and their corresponding receptors.

**TABLE 2:** Results of molecular docking of key targets and GRh3.

Targets	PDB ID	Compound	Binding energy (kcal/mol)
SRC	2SRC	GRH3	−9.2
ESR1	6SBO		−8.2
CASP3	4JJE		−8.1
EGFR	2G2		−7.6
MAPK1	2Y9Q		−8.5

### 3.5 The establishment of the insulin resistance model

**FIGURE 5:**
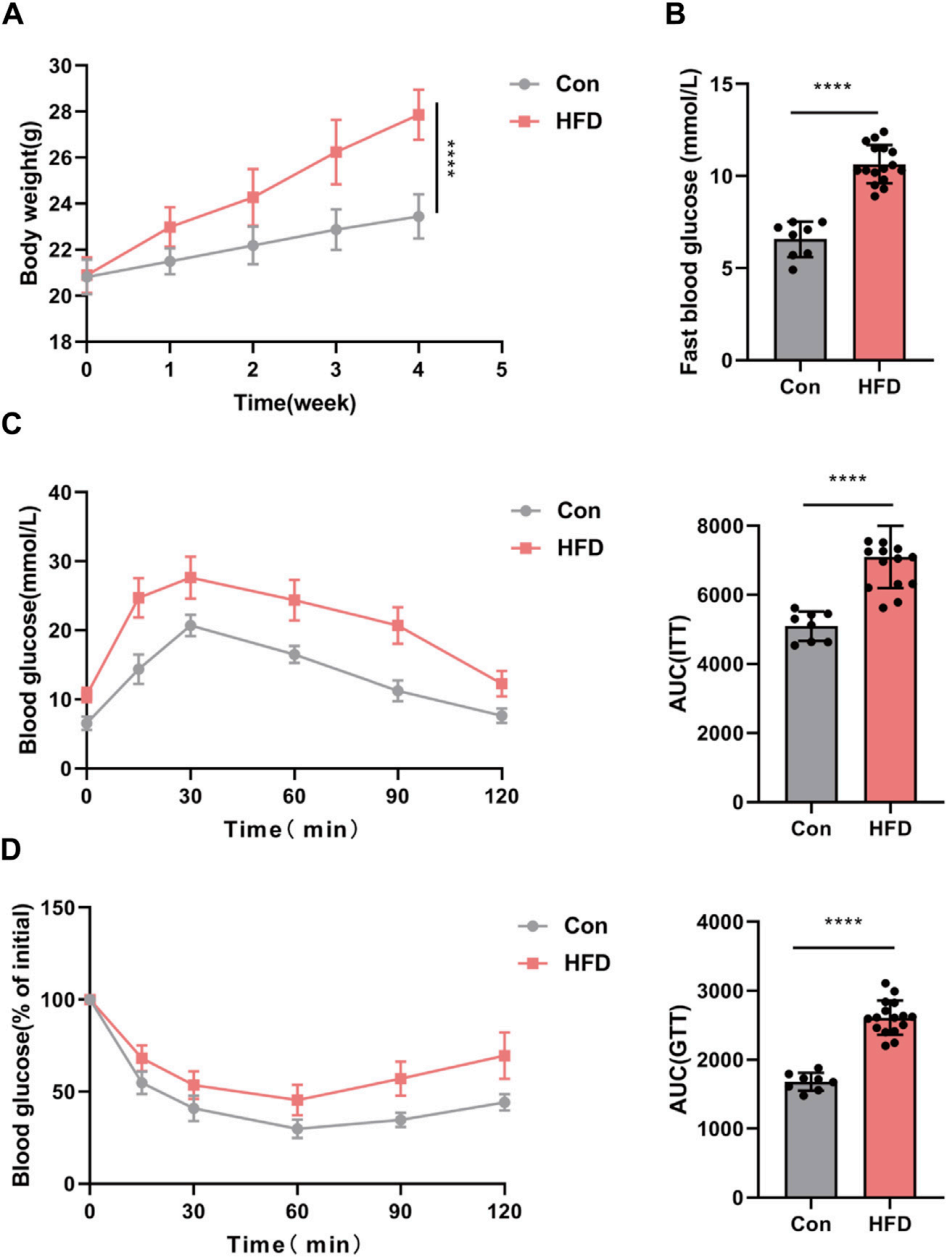
IR mouse model established with obese. **(A,B)** Body weight and fast blood glucose. GTT **(C)** and ITT **(D)** with the AUC conducted at 4 weeks (*n* = 6–8). *p < 0.05 or ****p < 0.001. Con, mice on a normal diet. HFD, mice on a high-fat diet (60% fat).

We have successfully established an insulin resistance mouse model to investigate obesity-induced insulin resistance *in vivo*. All mice were subjected to either a standard chow diet or a high-fat diet (HFD) for a duration of 4 weeks. Subsequently, following a 4-week intervention period, the HFD group displayed markedly greater body weight and elevated fasting blood glucose levels in comparison to the control group (Con), as depicted in Figures 5A, B.

To comprehensively evaluate glucose metabolism, glucose tolerance tests (GTT) and insulin tolerance tests (ITT) were conducted. The results of these tests unequivocally demonstrated impaired glucose tolerance in the HFD group, reaffirming the successful establishment of the insulin resistance mouse model (Figures 5C, D).

### 3.6 The effect of GRh3 on insulin sensitivity in high-fat diet-induced obese mice

**FIGURE 6:**
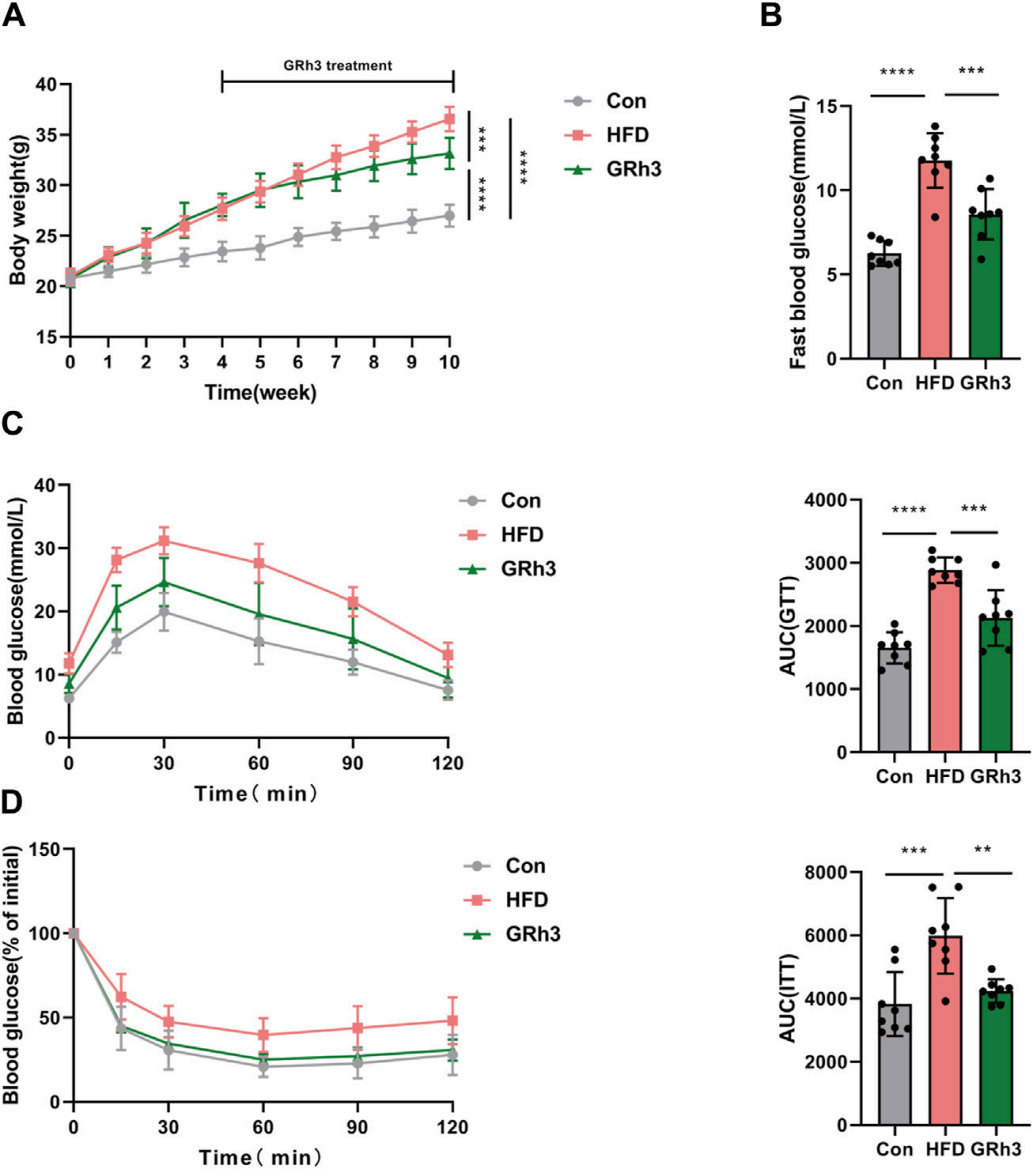
Effects of GRh3 on glucose tolerance and insulin sensitivity in obese mice. **(A,B)** Body weight and fast blood glucose of different groups at the end of the experiment. GTT **(C)** and ITT **(D)** with the AUC conducted at 10 weeks (*n* = 6–8). *p < 0.05 or ****p < 0.001.

In our pursuit to comprehend the impact of ginsenoside Rh3 (GRh3) on obesity in male mice, we meticulously organized our experimental cohorts. Mice initially assigned to the high-fat diet (HFD) group were stratified into two distinct groups, each comprising eight individuals: the HFD group itself and the GRh3-treated group (administered intraperitoneally at 10 mg/kg/day). Following an intervention spanning 6 weeks, discernible differences emerged. Specifically, the HFD group exhibited a significant escalation in body weight in contrast to the control group (Con). However, the administration of GRh3 distinctly attenuated the rate of body weight gain, concurrently demonstrating a reduction in fasting blood glucose levels among the HFD-induced obese mice (as illustrated in Figures 6A, B), and glucose tolerance and insulin sensitivity were significantly improved (Figures 6C, D).

### 3.7 GRh3 improves insulin resistance through modulating the expression of SRC, ESR1, EGFR, and MAPK1

**FIGURE 7:**
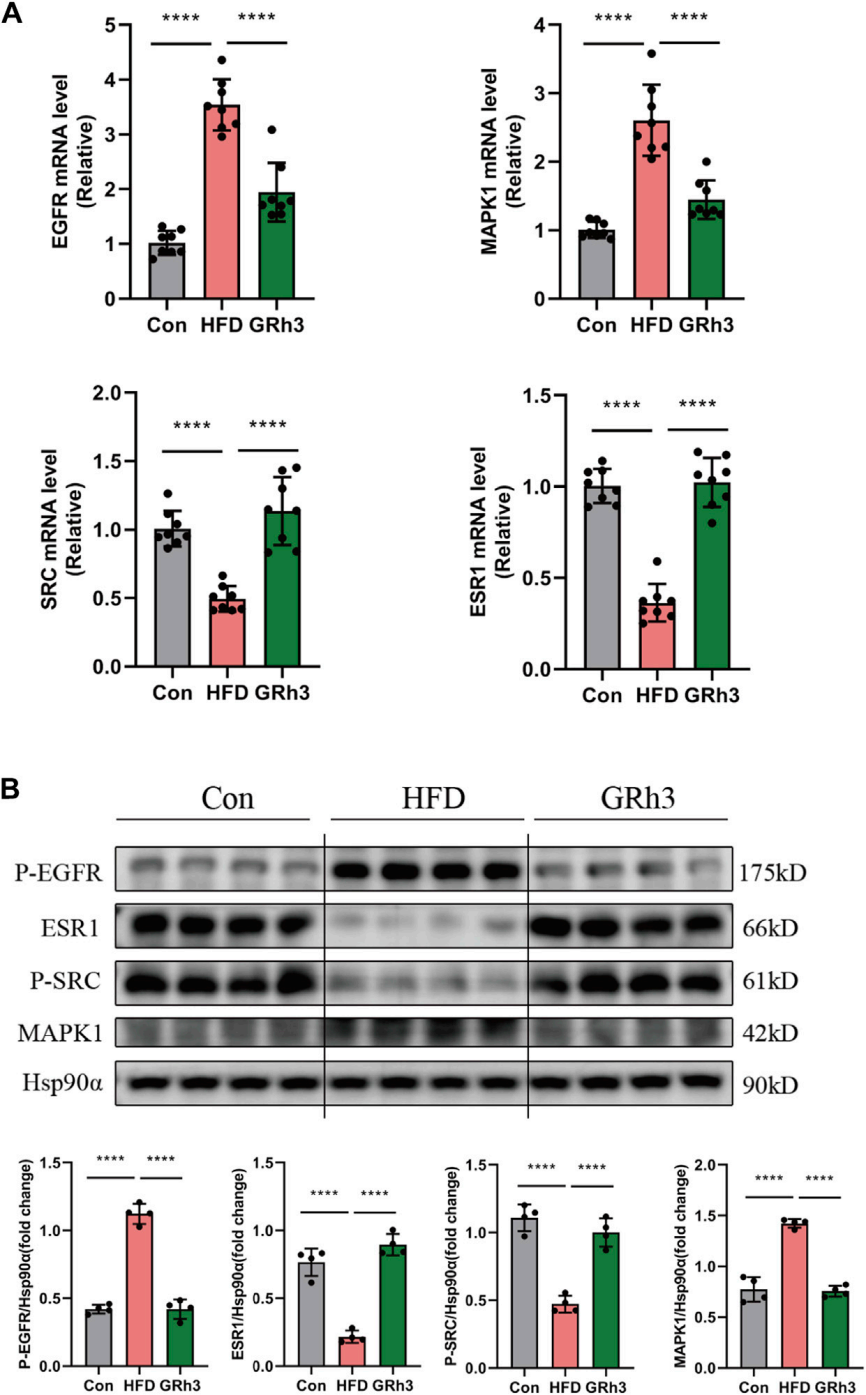
Effects of GRh3 on mRNA and protein expression of insulin resistance signaling pathway from the adipose tissue of mice in Con, HFD and GRh3 groups. **(A)** RT-PCR analysis of EGFR, ESR1, MAPK1, and SRC with GAPDH as reference control. **(B)** Western blot bands and result analysis of EGFR, ESR1, MAPK1, and SRC with Hsp90α as a reference control. *p < 0.05 or ****p < 0.001. The original images for blotsGels can be found in Supplementary Data Sheet 1.

In our quest to unravel the intricacies of how ginsenoside Rh3 (GRh3) mitigates insulin resistance induced by obesity, we conducted a multifaceted molecular analysis. Specifically, we isolated epididymal adipose tissue from the murine subjects and performed both reverse transcription-polymerase chain reaction (RT-PCR) and western blot analysis. Comparison of the results obtained from the GRh3-treated group with those from the control group (Con) yielded compelling insights. Notably, the relative mRNA levels of Phospho-EGFR and MAPK1 exhibited remarkable upregulation, while conversely, the levels of Phospho-SRC and ESR1 were notably downregulated in the Con group. Intriguingly, the intervention with GRh3 visibly counteracted this observed trend (as demonstrated in Figure 7A). The outcomes gleaned from the western blot analysis meticulously paralleled those discerned through RT-PCR (Figure 7B). This congruence lends further credence to the modulation of the SRC/ESR1/EGFR/MAPK1 signaling pathway as a pivotal mechanism through which GRh3 effectively ameliorates insulin resistance.

In conclusion, our findings substantiate that GRh3 exerts its insulin-sensitizing effects by intricately regulating the SRC/ESR1/EGFR/MAPK1 signaling pathway.

## 4 Discussion

Metabolic syndrome represented as obesity, T2D and NAFLD stands as a pervasive global health concern, often characterized as a silent epidemic. A defining characteristic shared among these conditions is the emergence of insulin resistance, which exacerbates their health ramifications. Consequently, the mitigation of insulin resistance emerges as a central tenet in the prevention and management of obesity and its associated metabolic sequelae (Tong et al., 2022).

In this study, ginsenoside Rh3 has been identified as a promising candidate for mitigating insulin resistance induced by a high-fat diet. Furthermore, our investigation has unveiled specific molecular targets, including EGFR, SRC, ESR1, and MAPK1, alongside critical signaling pathways such as insulin resistance, endocrine resistance, the FoxO signaling pathway, the PPAR signaling pathway, and the IL-17 signaling pathway, which elucidate the mechanisms underlying the ameliorative effects of GRh3 on insulin resistance. Employing an innovative approach, we have synergistically applied system pharmacology and bioinformatics analysis to predict the mechanisms of action of GRh3 in the treatment of insulin resistance. Moreover, this study represents the inaugural identification of potential targets and signaling pathways implicated in the mitigation of insulin resistance by GRh3 in obese mice.

Among the array of scrutinized signaling pathways, the IR pathway prominently emerges as a central player in the context of IR. Insulin’s physiological roles encompass stimulating glucose uptake and utilization, promoting lipogenesis, inhibiting glycogenolysis and gluconeogenesis, and preventing protein breakdown and lipolysis. However, in the face of metabolic burden, insulin signaling falters, fostering the development of IR. This phenomenon culminates in chronic systemic hyperinsulinemia, intricately linked to metabolic disorders (Huang et al., 2016; Zhang et al., 2020). Dysregulation of the insulin receptor and insulin signaling pathway transduction constitutes a pivotal factor underlying IR (Ramalingam et al., 2013).

Furthermore, the FoxO family of transcription factors assumes a significant role in the pathogenesis of IR. Notably, FoxO6 orchestrates insulin action on target genes and plays a critical role in the progression of hepatic glucose and lipid metabolism (Lee and Dong, 2017). Additionally, FoxOs exhibit multifaceted roles, either promoting IR or participating in the pathogenesis of obesity through the insulin/IGF-1 pathway (Homan et al., 2021).

The PPAR signaling pathway, another key regulator, intricately modulates insulin-related signal transduction and glucose metabolism (Chen et al., 2019). Activation of PPAR-γ has been shown to ameliorate the progression of liver macrovesicular steatosis and inflammation while enhancing glucose control by upregulating PPAR proteins in the hypothalamus (Diniz et al., 2021; Quarta et al., 2022).

IL-17, a pivotal regulator in energy homeostasis, assumes a crucial role in the interplay of insulin resistance, inflammation, and type 2 diabetes (T2D) (Olveira et al., 2023). Research into anti-IL-17 neutralizing antibodies has explored their potential to shield individuals at risk from T2D development (Abdel-Moneim et al., 2018).

In summary, our study underscores the intricate nexus between these pathways and the emergence and progression of IR in the context of metabolic disorders. Furthermore, it illuminates several promising avenues for targeted interventions in the treatment of IR.

Given the liver’s pivotal role as the major metabolic organ in regulating lipid metabolism and insulin action, we investigated the expression of insulin resistance-related molecules in the liver. In this study, we successfully established an obese insulin resistance mouse model through a high-fat diet (HFD), with its validation corroborated through rigorous assessments, including glucose tolerance tests (GTT) and insulin tolerance tests (ITT), as previously detailed (Xu et al., 2022). Our animal experimental findings unequivocally underscore the metabolic consequences of prolonged high-fat diet consumption. Mice subjected to extended high-fat diet regimens exhibited pronounced increases in body weight and fasting blood glucose levels, accompanied by exacerbated glucose intolerance and impaired insulin sensitivity when contrasted with the control group (Con). Notably, following a 6-week administration of GRh3, a discernible reduction in the rate of body weight gain among the obese mice was observed. Furthermore, GRh3 administration yielded a notable reduction in fasting blood glucose levels, along with improvements in glucose tolerance and insulin sensitivity. Collectively, these findings underscore the potential of GRh3 to ameliorate insulin resistance in the context of obesity. In this study, we have elucidated pivotal molecular targets underlying the amelioration of diabetic insulin resistance (IR) by GRh3. Notably, epidermal growth factor receptor (EGFR), estrogen receptor alpha (ESR1), SRC, and mitogen-activated protein kinase 1 (MAPK1) have emerged as central hub targets in this context.

EGFR, a member of the ErbB receptor family, exhibits a broad tissue expression pattern encompassing adipocytes, skeletal muscle, and hepatocytes (Berasain and Avila, 2014; Kim et al., 2021). Previous investigations have highlighted the upregulation of EGFR in macrophages during high-fat diet (HFD)-induced obesity. Selective macrophage-specific EGFR deletion has been shown to mitigate HFD-induced obesity, adipose tissue perturbations, and insulin resistance (Cao et al., 2022). Consistently, our animal study revealed elevated EGFR levels in HFD mice compared to controls, with GRh3 intervention effectively reversing this phenomenon.

ESR1 has been identified as a significant factor influencing Homeostatic Model Assessment for Insulin Resistance (HOMA-IR) in men, independent of confounding variables (Efstathiadou et al., 2015). Animal studies corroborate these findings, where global ESR1 knockout leads to reduced oxygen uptake, caloric expenditure, and heightened fasting insulin levels compared to wild-type counterparts. ESR1-deficient mice exhibit compromised glucose tolerance and marked insulin resistance (Ribas et al., 2010). Our *in vivo* experiments mirrored these outcomes, with HFD mice displaying significantly reduced ESR1 levels compared to controls, again rectified by GRh3 intervention.

Studies *in vitro* have highlighted the role of SRC in insulin resistance, with palmitate contributing to insulin resistance through inhibition of insulin-stimulated SRC phosphorylation and subsequent attenuation of SRC-mediated Akt phosphorylation (Feng et al., 2012). Consistently, ATXN2 knockout mice display insulin resistance, accompanied by reduced SRC levels in embryonic fibroblasts (Drost et al., 2013). Our study further substantiates the significance of SRC in insulin resistance, as we observed a marked reduction in SRC levels in HFD mice.

**FIGURE 8:**
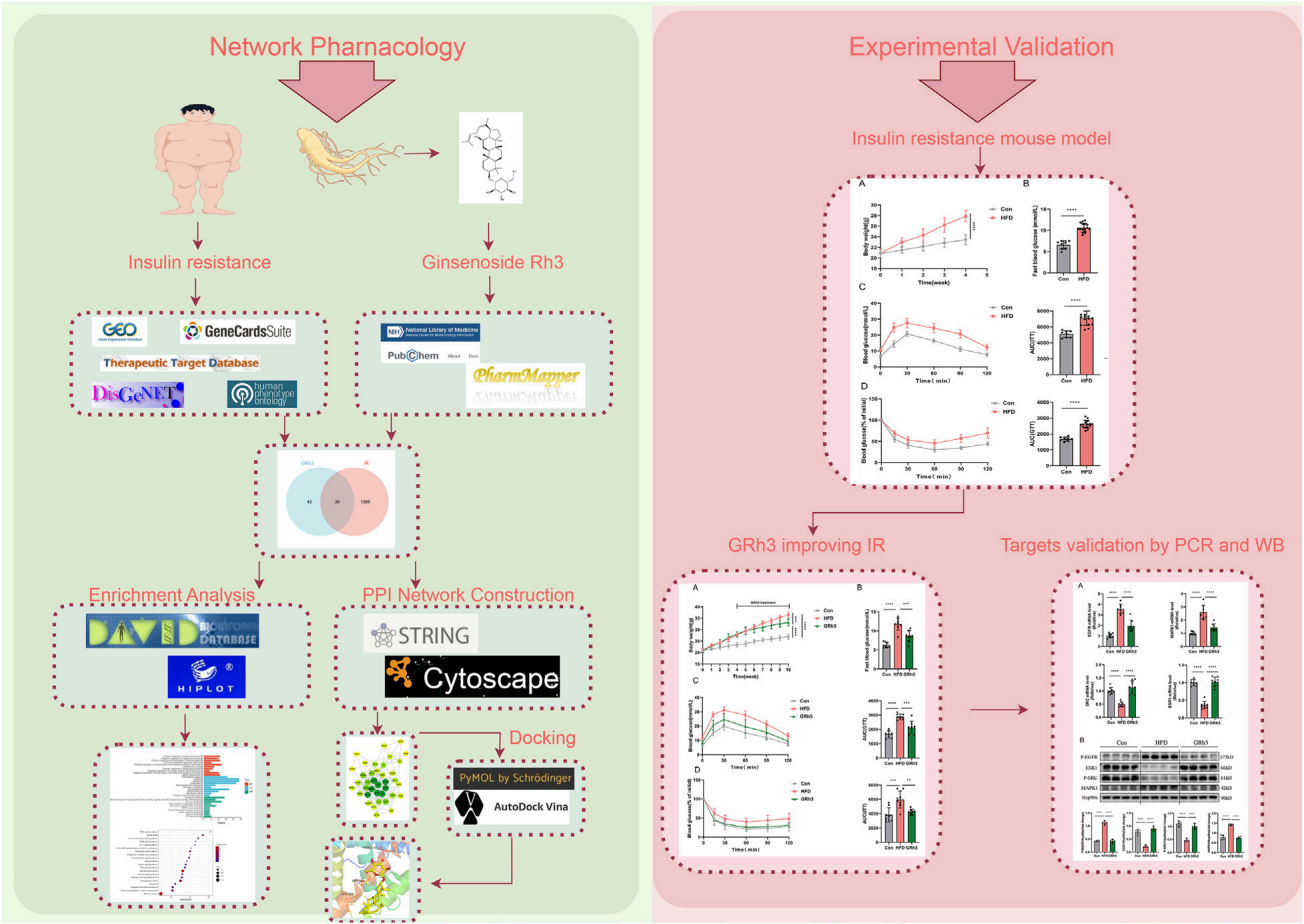
Flow chart of research. Detailed procedures of analysis are described in Materials and Methods. Briefly, The potential targets of GRh3 were obtained from PharmMapper database. IR-related targets were collected from GEO database, GeneCards, TTD, DisGeNet, and HPO databases. The potential targets of GRh3 in IR were generated by taking intersection of targets above. KEGG and GO enrichment analysis were performed using DAVID. STRING database and Cytoscape were used for the construction of PPI network analysis. The docking between GRh3 and key targets were conducted by PyMol and AutoDock. In vovo experiments were performed to verify the effect of GRh3 on IR and its underlying mechanism.

MAPK1, also known as extracellular signal-regulated kinase 2 (ERK2), a prominent member of the MAPK family, governs a spectrum of biological processes, including cell differentiation and transcriptional regulation, and is a major component of insulin signaling cascades. MAPK1 assumes a crucial role in the regulation of diabetes and inflammation (Ge et al., 2020). In mouse models of metabolic syndrome, ERK2 overexpression increased insulin resistance (Sato et al., 2022), while endothelial ERK2 deletion improved insulin resistance (Kujiraoka et al., 2022). Our results suggest that GRh3 may alleviate insulin resistance by downregulating MAPK1 expression. The flow chart of this research is illustrated in Figure 8. In summary, these central targets within the protein-protein interaction network underscore their pivotal roles in regulating obese insulin resistance. The effects of GRh3 on these specific targets offer fertile ground for future experiments and provide critical insights into the underlying mechanisms.

While our study has shed light on potential mechanisms of GRh3 against IR through the integration of system pharmacology and bioinformatics analysis, it is essential to acknowledge inherent limitations in our interpretation. Our study relies on the identification of differentially expressed genes (DEGs) through system pharmacology and bioinformatics analysis, which can be influenced by sample size accuracy. Thus, future investigations should consider expanding sample sizes to enhance the robustness and generalizability of our findings.

Furthermore, the absence of comprehensive experimental verification for the effects of GRh3 and the elucidated mechanisms through network analysis warrants attention. Addressing this deficiency is a focal point for our future research endeavors.

In conclusion, our study presents a promising avenue for understanding the potential mechanisms of GRh3 against IR. While acknowledging these limitations, they underscore the importance of further research aimed at enhancing the depth and applicability of our findings.

## 5 Conclusion

In conclusion, our comprehensive analysis unveils the potential targets and pathways associated with the amelioration of IR, specifically within adipose tissue, by GRh3. Our findings underscore the intricate nature of this mechanism, characterized by its multifaceted involvement across diverse targets, pathways, and mechanisms. This investigation serves as a robust cornerstone for future explorations, both in experimental research and potential clinical applications. We emphasize the imperative need for further molecular biological and pharmacological experiments to corroborate the functional significance of the identified key targets and underlying mechanisms.

## Data Availability

The datasets presented in this study can be found in online repositories. The names of the repository/repositories and accession number(s) can be found in the article/Supplementary Material.

## Abbreviations

IR, insulin resistance; GRh3, ginsenoside Rh3; DEGs, differentially expressed genes; PPI, protein-protein interaction; T2D, type 2 diabetes; TCM, Traditional Chinese Medicine; HFD, high-fat diet; GO, Gene Ontology; MF, molecular functions; CC, cellular components; FDR, False Discovery Rate; KEGG, Kyoto Encyclopedia of Genes and Genomes; GTT, glucose tolerance tests; ITT, insulin tolerance tests; RT-PCR, reverse transcription-polymerase chain reaction; EGFR, epidermal growth factor receptor; ESR1, estrogen receptor alpha; MAPK1, mitogen-activated protein kinase 1; HOMA-IR, Homeostatic Model Assessment for Insulin Resistance; GEO, Gene Expression Omnibus; non-IR, non-insulin resistance; STRING, Search Tool for the Retrieval of Interacting Genes; PDB, Protein Data Bank; SPF, specific pathogen-free; ipGTT, Intraperitoneal Glucose Tolerance Tests; ipITT, Intraperitoneal Insulin Tolerance Tests; qRT-PCR, Real-Time Reverse Transcription-Polymerase Chain Reaction.
