# Supplementary material for: Bioinformatics study of the potential therapeutic effects of ginsenoside Rh3 in reversing insulin resistance
Source: Front Mol Biosci. 2024 May 23;11:1339973. doi: 10.3389/fmolb.2024.1339973 (PMC11153663; doi:10.3389/fmolb.2024.1339973)
Supplement: Supplementary file 1 [file DataSheet1.docx]

Supplementary Material

***Original Images for BlotsGels***

**Full-length blot of Figure 7B protein expression level.**

**Figure S1.** Full-length blot of P-EGFR, MAPK1 and Hsp90α protein expression level. The following three membranes are cut from the same PVDF membrane.


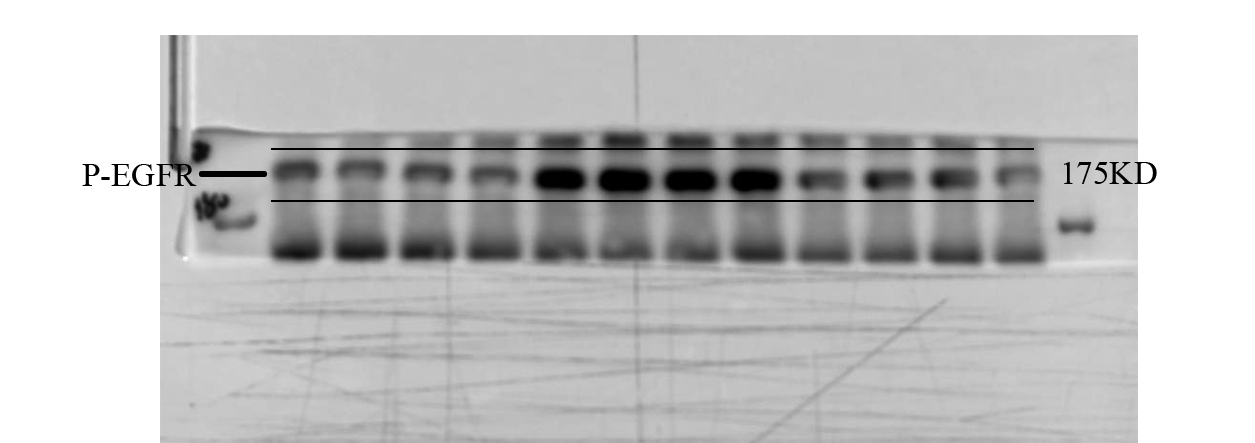

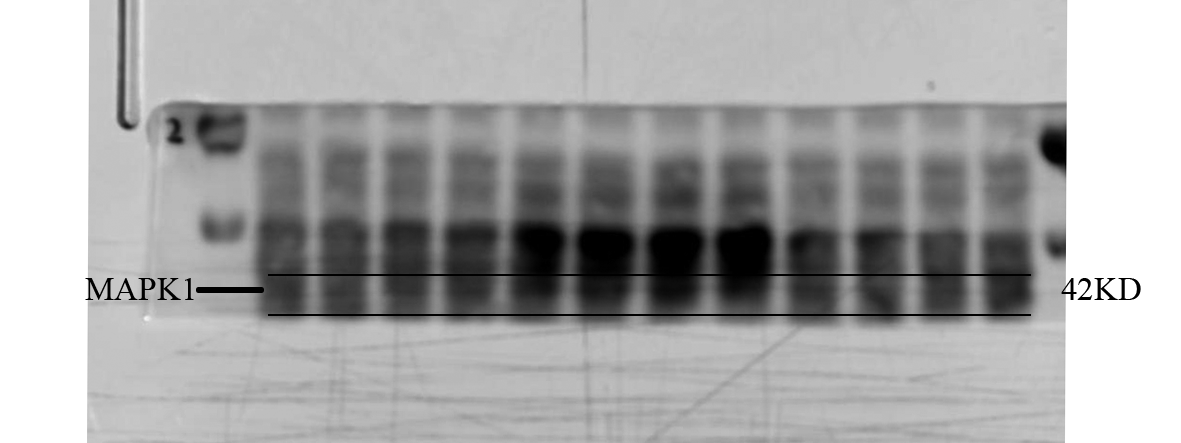


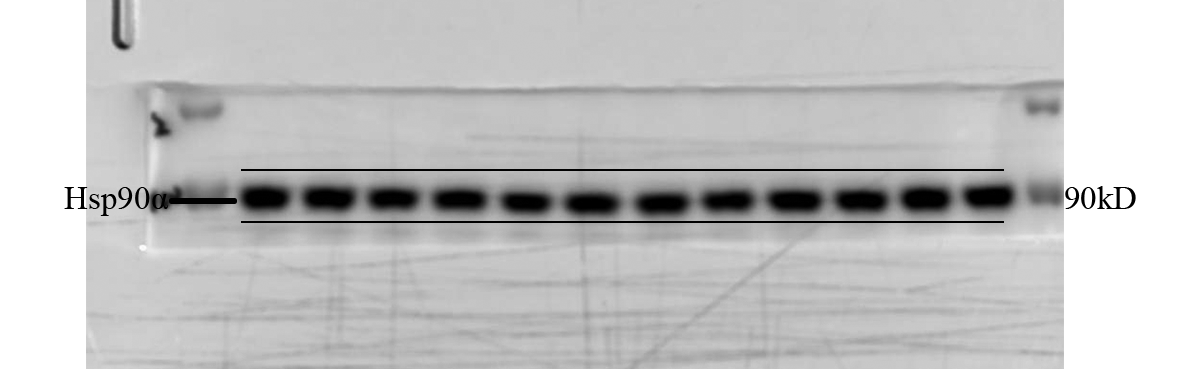


**Figure S2.** Full-length blot of ESR1 and Hsp90α protein expression level. The following two membranes are cut from the same PVDF membrane.


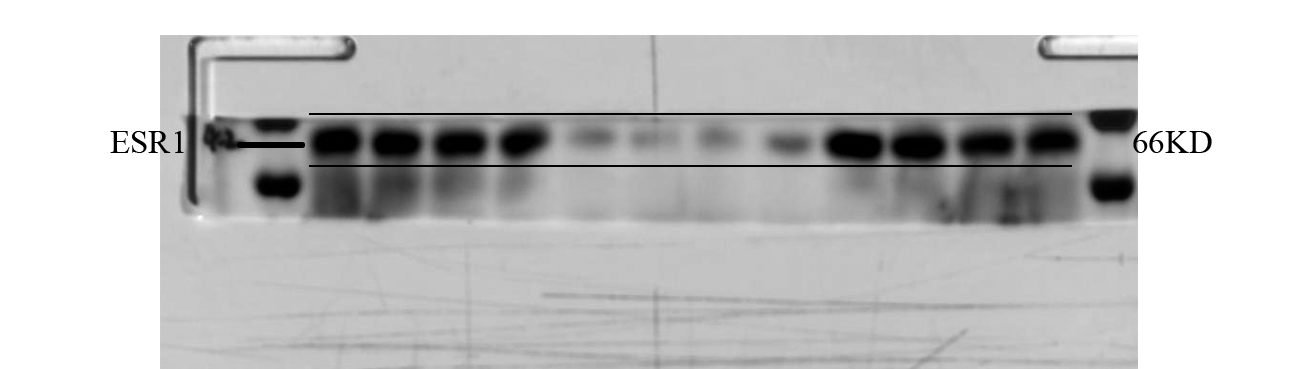


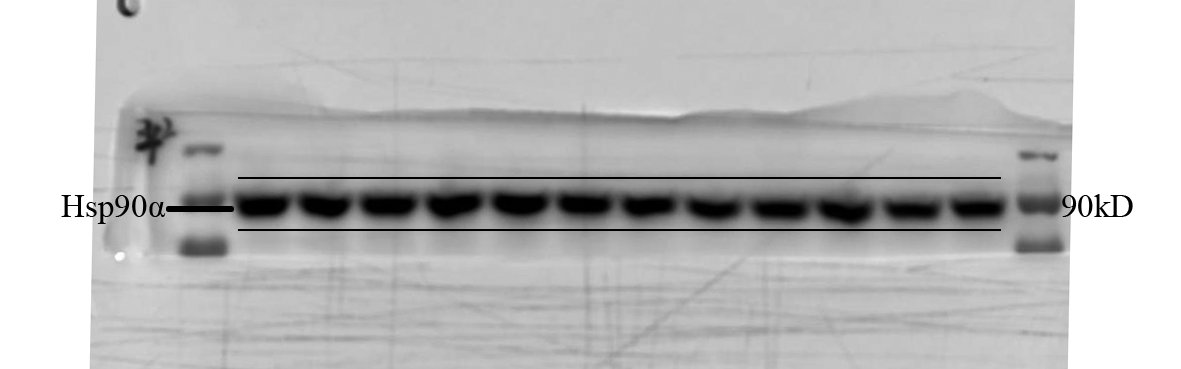


**Figure S3.** Full-length blot of P-SRC and Hsp90α protein expression level. The following two membranes are cut from the same PVDF membrane.


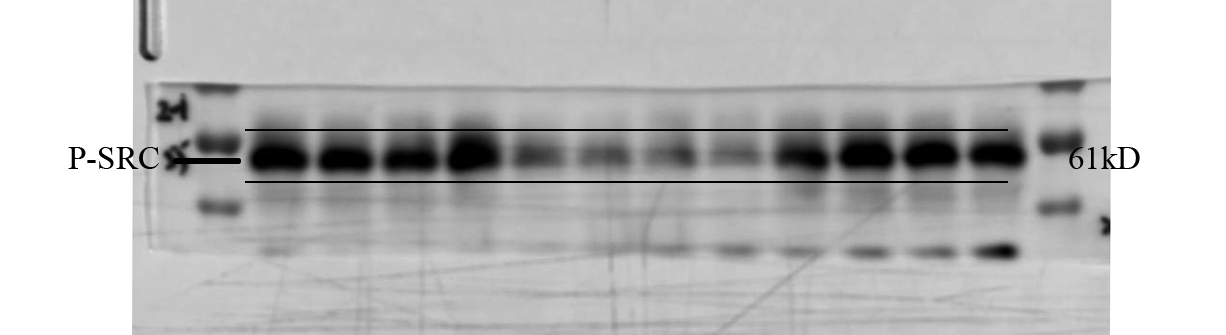


**
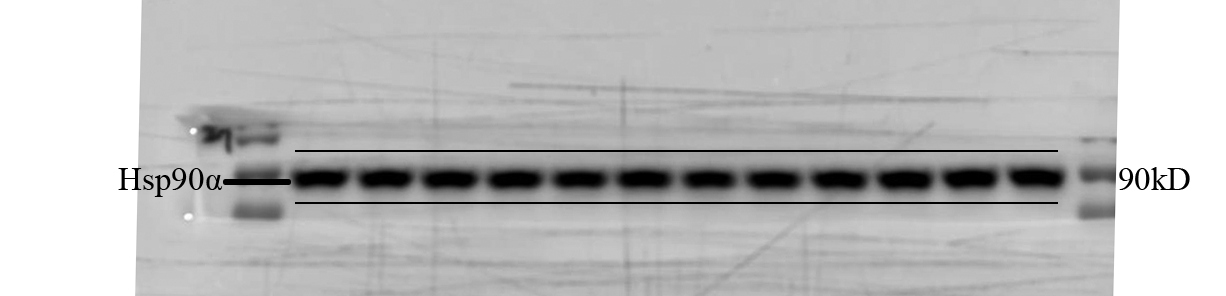
**
